# A Comprehensive Study of Gemfibrozil Complexation with β-Cyclodextrins in Aqueous Solution Using Different Analytical Techniques

**DOI:** 10.3390/ijms232416119

**Published:** 2022-12-17

**Authors:** Arantza Zornoza, Itziar Vélaz, Gustavo González-Gaitano, María Cristina Martínez-Ohárriz

**Affiliations:** Department of Chemistry, School of Sciences, University of Navarra, c/Irunlarrea s/n, 31008 Pamplona, Spain

**Keywords:** cyclodextrins, inclusion complexes, fluorescence, NMR spectroscopy, solubility isotherms, thermodynamic parameters

## Abstract

Gemfibrozil (GEM) is a hypolipidemic agent, which is effective in reducing serum cholesterol and triglyceride levels. Complexation of GEM with native β-cyclodextrin (β-CD) and with the derivatives hydroxypropyl-β- and randomly methylated β-CD (HPβ-CD and Meβ-CD) was studied in aqueous solution of pH 2.8 and 7.0. The stability constants were determined by spectrofluorimetry, ^1^H-NMR spectroscopy and solubility assays. Considering the well-known difficulties to obtain similar stability constants by different techniques, the agreement of the values obtained supports the reliability of the results presented. The advantages and drawbacks of each analytical technique for the study of inclusion complexation were discussed as well. In addition, the thermodynamic parameters of complexation, enthalpy (ΔH) and entropy (ΔS), were determined and related to the type of molecular interactions that take place between GEM and the different cyclodextrins. Finally, solid dispersions were prepared by co-evaporation, kneading, vacuum desiccation, and coprecipitation, and complexation was evaluated by X-ray diffraction.

## 1. Introduction

Cyclodextrins are cyclic oligosaccharides with the ability to form inclusion complexes due to their toroidal shape, which exhibits a relatively hydrophobic cavity and a hydrophilic outer surface. These complexes can be used to improve the solubility, bioavailability, and stability of drugs [1,2,3,4]. The cavity size of β-cyclodextrin (β-CD) is optimal for complexation of benzene derivatives. The substitution of β-CD hydroxyl groups involves changes in the rim polarity, cavity volume, and in the molecular flexibility, which can tune the supramolecular interactions with a specific guest [5]. The derivatives methyl-β-CD (Meβ-CD) and hydroxypropyl-β-CD (HPβ-CD), more soluble than the parent compound, are of pharmaceutical interest and both have been marketed in different formulations [6].

**Figure 1:**
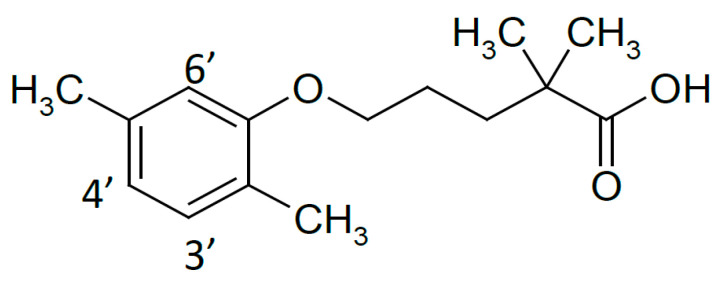
Chemical structure of gemfibrozil (GEM).

Gemfibrozil (GEM), 5-(2,5-dimethylphenoxy)-2,2-dimethylpentanoic acid (Figure 1), a benzene derivative of valeric acid, is a hypolipidemic agent effective in reducing serum cholesterol and triglyceride levels. It is a non-hygroscopic lipophilic drug whose poor solubility and low dissolution rate in the gastrointestinal tract limits its bioavailability after oral administration. Several methods have been used to improve its dissolution characteristics, such as micronization [7], solubilization by lipids, and complexation with γ-CD [8] and dimethyl-β-CD [9,10]. The physicochemical characteristics of this drug determine its suitability to achieve a bioavailability enhancement through complexation with β-CD [11].

It is well known that studies of complexation with cyclodextrins often lead to discordant values of stability constants depending on the experimental conditions, the analytical technique employed, and the mathematical model used to analyse the experimental data. This is the case of the complex gemfibrozil-β-CD [10,12].

The present paper is a comprehensive investigation of the complexation of GEM with native β-CD and with the derivatives hydroxypropyl-β- and randomly methylated β-CD at pH 2.8 and 7.0. These derivatives were selected with the aim of evaluating the effect of the polarity of the substituents on the complex stability. The analysis of complexation, which comprises spectroscopic methods (spectrofluorimetry and nuclear magnetic resonance, NMR) as well as solubility isotherms, focused on two aspects: to obtain reliable values of the binding constants by using different yet complementary methods and to ascertain the factors that lead to discrepancies between different experimental methods. The non-inclusion solubilizing effects of cyclodextrins [13,14] were considered in the interpretation of the solubility isotherms. In addition, solid dispersions were prepared by co-evaporation, kneading, vacuum desiccation, and coprecipitation, and the solid-state complexation was evidenced by X-ray powder diffraction.

## 2. Results and Discussion

### 2.1. Nuclear Magnetic Resonance (^1^H-NMR) Spectroscopy

The formation of the complex GEM-β-CD was studied by ^1^H NMR spectroscopy at 22 °C. The low solubility of the drug in acid medium precluded any quantitative study, but it was possible to determine the stability constant at pH 10.0.

**Figure 2:**
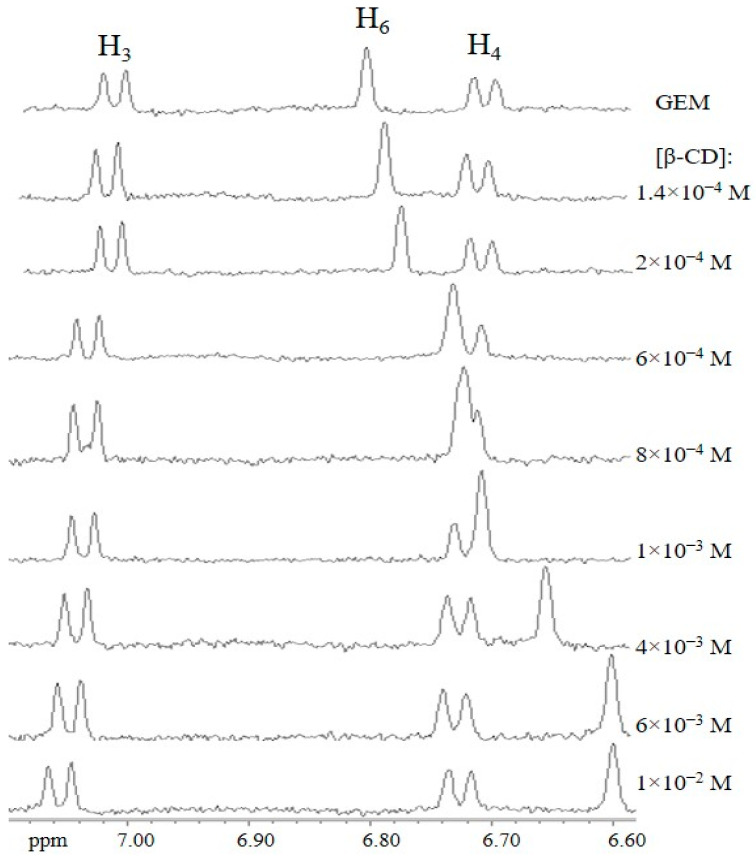
^1^H-NMR spectra of the aromatic region of gemfibrozil in the presence of increasing concentrations of β-CD at pH 10.

The NMR spectra of the aromatic region of GEM in the presence of increasing concentrations of β-CD at pH 10.0 are shown in Figure 2. The aromatic protons of GEM, H3, and H4, shifted upfield, whereas H6 experiences a strong downfield shift (Δδ = −0.21 ppm, over the range 0 to 10 mM of macrocycle). The methyl protons of the aromatic ring also shifted upfield, although to a lesser extent (maximum Δδ = −0.06). On the other hand, the cavity protons of the β-CD, H3′, and H5′, underwent significant shifts. This evidence proves the inclusion of the aromatic ring of the drug in the macrocycle cavity. It is reasonable to assume that the mode of GEM binding to HP-βCD and Me-βCD will also involve the aromatic ring, as the cavity is similar.

Microscopic stability constants were deduced by non-linear squares fitting of selected protons of both host and guest according to the procedure described in Materials and Methods. The good fits obtained support the 1:1 stoichiometry of the complex at this pH. By using the H6 chemical shifts of the GEM, a *K*_1:1_ value of (9.7 ± 0.6) × 10^2^ M^−1^ was obtained. The same procedure applied to the H3′ and H5′ protons of the β-CD yielded values of (9.7 ± 1.1) × 10^2^ M^−1^ and (11.1 ± 1.7) × 10^2^ M^−1^, respectively.

### 2.2. Fluorescence Spectroscopy

Complexation of GEM with β-, Meβ- and HPβ-CD was studied at different temperatures in aqueous solutions at pH 2.8 and pH 7.0, where the drug was in its neutral and ionized form, respectively (pKa = 4.7).

First, the fluorescence quantum yields of gemfibrozil alone and in the presence of β-CDs at 25 °C were determined at pH 2.8 and pH 7.0 (Table 1). The GEM fluorescence in aqueous solution was almost pH-independent. An increase in the fluorescence quantum yield of the drug was observed in the presence of all the cyclodextrins studied. This enhancement can be applied to improve the fluorimetric analysis of this drug in aqueous media. The values obtained for the quantum yields with β-CDs were higher than those of the complexes with γ-CDs [8], most likely because the smaller size of the cavity yields a better host-guest fitting.

**Figure 3:**
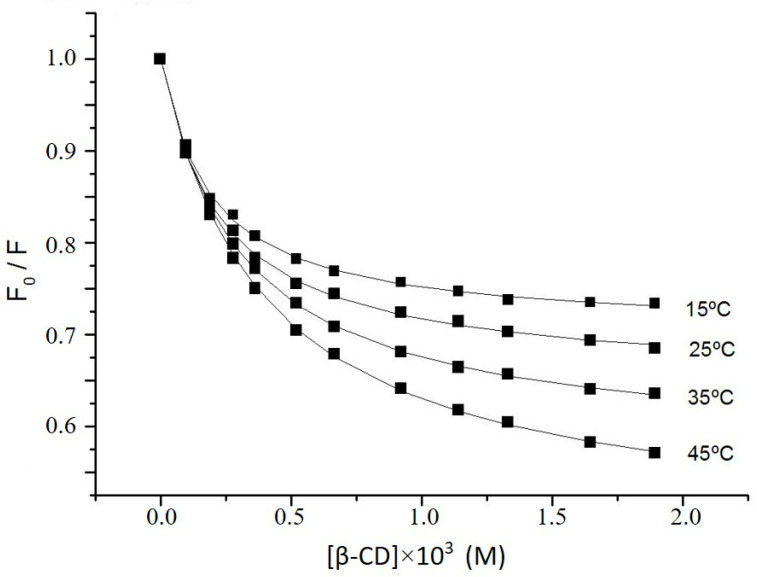
Plots of the fluorescence data of gemfibrozil (Equation (2)) in the presence of β-CD at pH 2.8 and different temperatures (λexc = 272 nm; λem = 303 nm).

The fluorescence enhancement of gemfibrozil in the presence of increasing concentrations of β-CDs can be used to determine the stability constants of the complexes. Figure 3 shows, as an example, the results of the assay with β-CD at pH 2.8. This fluorescence enhancement can be explained by the supramolecular interaction between the guest and the CDs, which involves the inclusion of the phenoxy group of gemfibrozil in the host cavity, as has been proved by NMR spectroscopy. This increase of fluorescence upon inclusion in the CD cavity can be ascribed to the protection of the excited state of the guest against external deactivators and to a restriction of its molecular degrees of freedom.

In order to determine the binding parameters and to confirm the 1:1 stoichiometry, the values of F_o_/F versus cyclodextrin concentration [CD] were fitted to Equations (2) and (3) at different temperatures (Figure 3). The fits are consistent with the assumed 1:1 stoichiometry and enable the determination of K_11_ and values at each temperature (Table 2).

With respect to the *a* values defined by the ratio ϕ_c_ε_c_/ϕ_g_ε_g_, they increased at high temperatures as a consequence of a lower influence of collisional deactivation of the complex in comparison with the free drug. As the band at the longest wavelength in the absorption spectrum of gemfibrozil does not change in the presence of β-CDs, the molar absorptivity of the complex (ε_c_) and the free guest (ε_g_) are nearly the same at the excitation wavelength and the *a* value becomes a ≈ ϕ_c_*/*ϕ_g_. From the temperature dependence of *a* and considering that the fluorescence of GEM decreases markedly with temperature (approximately 0.8% per °C [15]), it is possible to infer a protective effect of the excited state of the fluorophore upon inclusion. Finally, it was found that the ratios ϕ_c_*/*ϕ_g_ obtained from the *a* values were similar to those calculated from the experimental data of quantum yields previously shown in Table 1.

In relation to the binding parameters collected in Table 2, remarkably higher binding constants were obtained at pH 2.8 with all the CDs studied. In this medium, gemfibrozil was neutral (pKa 4.7) and the supramolecular interactions with the relatively non-polar cavity of the CD are favoured. The highest K_1:1_ values were found with Meβ-CD, remarking the favourable influence of the methyl substituents for the complex stability with the neutral form of the drug. The binding constants with β- and HPβ-CD at pH 2.8 were quite similar, especially at high temperatures. By contrast, at pH 7.0, β-CD provides the highest stability constant, the lowest value being that of the complex with Meβ-CD. This evidence is once more in accordance with the less polar nature of Meβ-CD rims, which make the inclusion of an ionized guest less favourable. The binding constant obtained with β-CD by fluorescence spectroscopy at pH 7.0 and 25 °C was similar to the one obtained by H-NMR spectroscopy at 22 °C and pH 10 as in both experiments GEM was ionized.

The K_1:1_ obtained for the complex GEM-β-CD at 25°C in pH 7.0 aqueous solution (7.6 ± 0.3) × 10^2^ M*^−^*^1^ agrees with the value of 757 M*^−^*^1^ reported for the same complex at pH 9.1 by Tang et al. (2004) [16], contrasting with the higher values obtained by Huang et al. (2007) [12] in the same conditions (around 3000 M*^−^*^1^).

Regarding the thermodynamic parameters (Table 3), the enthalpies of complexation, as obtained by the Van’t Hoff equation, correspond to an exothermic process, as is usual in CD complexes. The highest negative values were obtained for the complex with β-CD, while the complexation with Meβ-CD produced the lowest energy released. This suggests the contribution to the binding of stronger interactions, such as H-bonds, more intense and directional with β-CD than with the substituted macrocycles.

It is well known that the stability of the H-bond decreases with temperature [17]. In a first approximation, the lower stability of the systems with β-CD at high temperatures could be attributed to the rupture of these bonds. The role of H-bonding in the complex stability seems also supported by the higher enthalpy obtained with the β-CD at pH 2.8, conditions in which the carboxylate group of GEM is in its protonated form. In addition, the negative entropy of the β-CD complex at pH 2.8 reflects a decrease of the degrees of freedom when the H-bonds are established.

In summary, the thermodynamics of complexation is typical of inclusion complexes with CDs, i.e., exothermic binding in which the substitution of the β-CD by hydroxypropyl and methyl groups reduces the enthalpic contribution but increases the entropic one to the free energy of binding.

### 2.3. Solubility Isotherms

**Figure 4:**
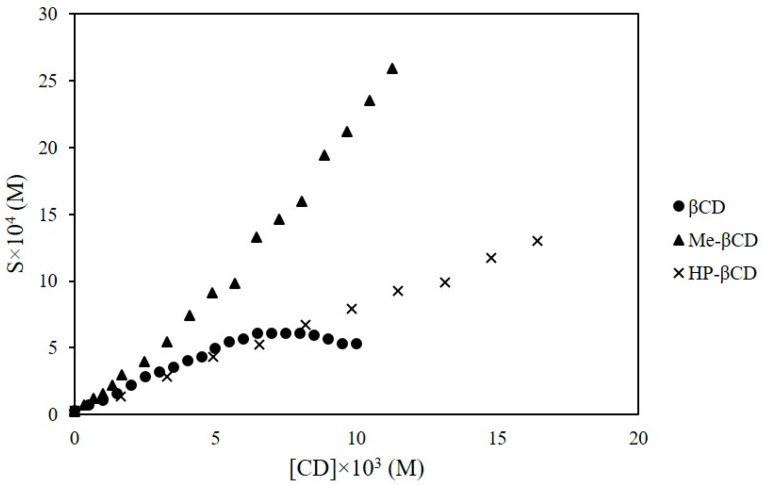
Solubility isotherms of gemfibrozil in the presence of different cyclodextrins at pH 2.8 and 25 °C.

The solubility of GEM determined in pH 2.8 aqueous solution at 25 °C was (3.2 ± 0.3) × 10*^−^*^5^ M. The influence of β-, Meβ-, and HPβ-CD concentration on the solubility of GEM in the same experimental conditions is depicted in Figure 4.

The complexation of GEM with β-CD gave rise to a Bs type solubility isotherm that corresponds to a complex with limited solubility. Initially, there was a linear increase of GEM solubility with the concentration of β-CD until a plateau was reached from 6.0 × 10*^−^*^3^ M β-CD. Up to this point a 20-fold increase of GEM solubility was achieved. The molar stoichiometry of the complex can be determined from the extension of the plateau region (Equation (8)). The obtained stoichiometry of the GEM/β-CD complex was 1:1. The stoichiometry with respect to the ligand (CD) was confirmed from the plot of ln[(St − So)/So)] versus ln[β-CD] in the initial linear part of the isotherm (Equation (5)). Therefore, this is the stoichiometry assumed for the determination of the stability constant of the complex, which was estimated from the slope and the intercept of the initial linear portion of the isotherm (Equation (7)) and resulted in (3.2 ± 0.3) ×·10^3^ M*^−^*^1^ (R^2^ = 0.998).

The solubility isotherm of the complex with Meβ-CD presented an initial linear region followed by a positive deviation from linearity (A_P_ type). This profile is usually associated to the formation of both 1:1 and 1:2 GEM-Meβ-CD complexes. The values obtained by fitting the experimental data to Equation (9) were K_1:1_ = (4.0 ± 0.2)·× 10^3^ M*^−^*^1^ and K_1:2_ = 71 ± 15 M*^−^*^1^.

The solubility of GEM increases linearly with the concentration of HPβ-CD (A_L_ type isotherm). A 1:1 stoichiometry with respect to the ligand was determined from the plot of ln[(St − So)/So)] versus ln [HPβ-CD] (Equation (5)). The stability constant, K_1:1_ = (2.6 ± 0.1) × 10^3^ M*^−^*^1^, was determined from the slope and the intercept obtained from the regression analysis. It is worth mentioning the good agreement between the values obtained by solubility isotherms and by fluorescence spectroscopy.

The highest solubility enhancement, a 90-fold increase, was achieved with 1.2 × 10*^−^*^2^ M Meβ-CD, whereas the same concentration of HPβ-CD produced a 30-fold. Both β-CD derivatives present surface activity [18], which could contribute to the solubilisation of the drug to a certain extent.

In relation to other solubility studies carried out with related hypolipidemic agents, Bs type isotherms with natural cyclodextrins and A_L_ type with CD derivatives were reported for clofibrate [19] and for fenofibrate with HPβ-CD [20].

### 2.4. Remarks on the Suitability of the Different Experimental Techniques Used to Determine the Binding Constants of Cyclodextrins Complexes in Solution

There are some considerations to point out with respect to the different techniques available to study inclusion complexation in aqueous solution. If the guest molecule is fluorescent and its solubility is poor, as in the case of GEM, the elected technique is fluorescence spectroscopy due to its high sensitivity. ^1^H-NMR spectroscopy presents important limitations in this case because its lower sensitivity makes it necessary to use higher amounts of both guest and cyclodextrins. However, when the solubility is not an issue, ^1^H-NMR spectroscopy achieves the double goal of determining the stability constant together with valuable information on the specific interactions occurring between host and guest, which may permit, in certain cases, to gain insight into the 3D structure of the complex. The solubility assays are a very interesting option for poorly soluble substrates that cannot be analysed by spectroscopic methods. It should be remarked that only the very first part of the solubility isotherms ensures the absence of non-inclusion effects [13], such as aggregation or formation of supersaturated drug solutions [14,21]. Likewise, the agreement between the solubility of the pure guest and the value of the intercept of the fitting should be used to verify the consistency of the method.

### 2.5. Solid State Complexation

**Figure 5:**
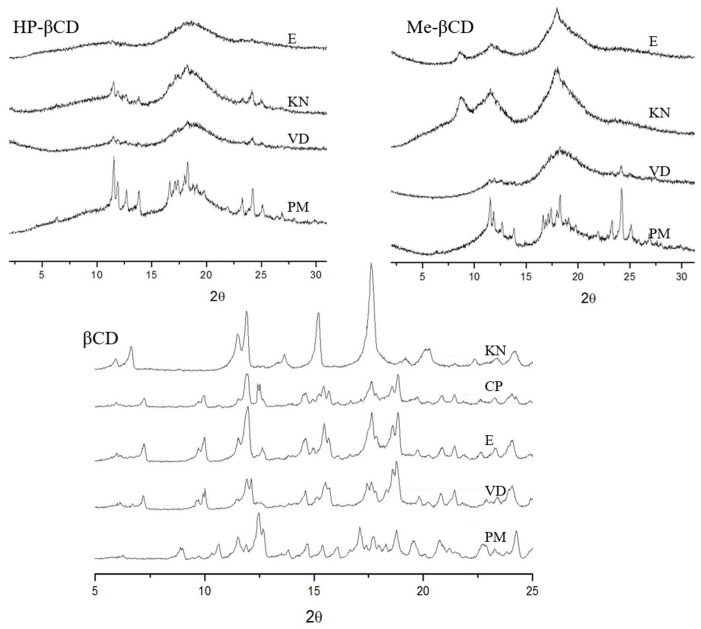
X-ray diffraction pattern of the physical mixtures (PM) and the complexes formed by kneading (KN), coprecipitation (CP), co-evaporation (E) and vacuum desiccation (VD).

Figure 5 shows the X-ray diffraction analysis (XRD) of all the solid dispersions prepared by co-evaporation, vacuum desiccation, kneading, and coprecipitation. The GEM-β-CD physical mixture shows the overlapping of the diffraction peaks corresponding to both GEM (2θ = 11.5, 11.9, 12.7, 13.8, 16.6, 17.4, 18, 18.3 and 24.2°) and β-CD (9.1, 12.5, 12.7, 17.0, and 22.5°). The diffraction patterns of GEM-βCD systems are different from that of the physical mixture, suggesting the formation of complexes in the solid state. The co-evaporated, coprecipitated, and vacuum desiccated systems present a similar profile, which is at the same time different from that of the kneaded system. These diffraction patterns suggest the formation of two different crystalline structures for the complexes depending on the preparation method.

However, the physical mixtures of GEM with Meβ-CD and HPβ-CD present a diffraction pattern with the peaks of the drug overlapped with the amorphous profile of the corresponding cyclodextrin. The reflections of GEM disappeared from all the solid dispersions obtained with Meβ-CD due to complexation. In the case of solid phases GEM-HPβ-CD, an amorphous complex is obtained by co-evaporation and vacuum desiccation and only partial complexation is achieved by kneading.

## 3. Materials and Methods

### 3.1. Materials

Gemfibrozil was purchased from Sigma (Madrid, Spain). β-CD was from Roquette S.A. (Lestrem, France), Meβ- (DS ≈ 12) and HPβ-CD (DS ≈ 4,5) from Cyclolab (Budapest, Hungary). All other reagents and solvents were from Panreac (Barcelona, Spain).

### 3.2. Methods

#### 3.2.1. ^1^H Nuclear Magnetic Resonance (^1^H-NMR) Spectroscopy

A Bruker Avance 400 Ultrashield NMR spectrometer (Germany) was employed. The measurements were carried out at 295 K in D_2_O containing NaOD (pH 10). The chemical shifts were determined taking as the reference the HDO signal. The concentration of gemfibrozil was 3.8 × 10^−^^4^ M and that of β-CD ranged from 0 to 1 × 10^−^^2^ M. The ^1^H-NMR spectra of sets of solutions containing the same concentration of GEM and different concentrations of β-CD were recorded at pH 10.0 because of the poor solubility of the drug at lower pH values.

The stability constant of the complex can be calculated provided that the complexation is fast in the NMR time scale. The chemical shift measured for a certain proton (δ) is the weighted average of the molecules in its different forms. For example, assuming 1:1 complexation, if the chemical shift observed (δ) corresponds to the substrate:

(1) $$\delta =X_{S}\delta_{S}+X_{SL}\delta_{SL}$$

where $X_{S}$ and $X_{SL}$ are the molar fractions of free substrate (either host or guest) and complex, and δ_s_ and δ_SL_ are their respective chemical shifts. The concentrations of all the species, connected by the mass balance and mass action law, were obtained by nonlinear least squares fitting of the measured chemical shifts versus the concentration of either host or guest protons [22], using Origin Pro 8.5 software.

#### 3.2.2. Fluorescence Spectroscopy

Steady-state fluorescence experiments were performed using a LS50 Perkin–Elmer spectrofluorimeter (Perkin Elmer, Rodgau, Germany). The fluorescence quantum yields were measured using optically diluted solutions (absorbances lower than 0.01), by comparison of the corrected emission spectra of gemfibrozil with that of quinine bisulphate in 0.1 N sulphuric acid [23], the excitation wavelength employed was 260 nm and the slit width 2.5 nm.

The binding constants between gemfibrozil (pKa 4.7) and β-CDs were calculated at pH 2.8 and 7.0. These pH values were obtained from HCl and phosphate buffer solutions, respectively. In each titration, gemfibrozil concentration was 8.0 × 10^−^^6^ M and β-CD concentrations increased from 0 to 2.0 × 10^−^^3^ M. The experimental conditions were λexc = 272 nm, λem = 303 nm, slit widths = 2.5 nm.

The experimental data obtained were fitted to the following equation, which assumes a 1:1 stoichiometry [24]:

(2) $$\frac{F_{0}}{F}=\frac{1+K_{1:1}\left[CD\right]}{1+aK_{1:1}\left[CD\right]}$$

where $F_{0}$ is the fluorescence intensity of free gemfibrozil, *F* represents the intensity in the presence of CDs, $K_{1:1}$ is the binding constant and *a* parameter is defined by: *a = ϕ_c_ε_c_/ϕ_g_ε_g_*, where *ε* and *ϕ* are the molar absorptivities and fluorescence quantum yields of the complex (c) and the free guest (g), respectively. The linear form of Equation (2) was used to calculate *K*_11_ and *a* values:

(3) $$\frac{\left[CD\right]}{1-\frac{F_{0}}{F}}=\frac{1}{\left(a-1\right)K_{1:1}}+\frac{a}{a-1}\left[CD\right]$$

The thermodynamic parameters enthalpy (ΔH) and entropy (ΔS) of complexation were determined from the temperature dependence of the binding constants according to the Van’t Hoff equation, assuming these magnitudes remain constant within the temperature range studied:

(4) $$lnK_{1:1}=-\frac{\Delta H}{RT}+\frac{\Delta S}{R}$$

#### 3.2.3. Solubility Isotherms

The solubility experiments were carried out in pH 2.8 aqueous solution by adding amounts of 20 mg amounts of gemfibrozil to test tubes containing 20 mL of increasing concentrations of CD, ranging from 0 to 1.6 × 10^−^^2^ M. These tubes were sonicated for 1 h and then kept in a shaking water bath at 25 °C and 80 rpm until equilibrium was reached (14 h approximately). Samples were then taken by filtration (0.45 μm pore filters) and measured at 216 and 274 nm using a HP8452A diode-array spectrophotometer (Madrid, Spain). When required, the samples were diluted to obtain absorbance values lower than 0.9. The assays were performed in triplicate.

The apparent stability constant and stoichiometry of the complexes can be deduced from the phase solubility diagrams by plotting the solubility of the drug versus the CD concentration [24]. The stoichiometry of the complex with respect to the ligand (CD) can be estimated from the slope of the equation:

(5) $$ln\frac{S_{t}-S_{0}}{S_{0}}=n\;ln\left[CD\right]+lnK_{1:n}$$

where $S_{0}$ is the solubility of pure gemfibrozil, $S_{t}$ is the solubility at a given CD concentration and K_1:n_ is the apparent stability constant of the 1:n complex.

The equation that describes linear diagrams, named A_L_ by Higuchi and Connors [25], is:

(6) $$S_{t}=S_{0}+\frac{K_{1:1}S_{0}\left[CD\right]}{1+K_{1:1}S_{0}}$$

where $K_{1:1}$ is the apparent stability constant of a 1:1 complex. The stability constant can be determined from the slope and the intercept of the linear solubility diagram.

(7) $$K_{1:1}=\frac{m}{S_{0}\left(1-m\right)}$$

B type solubility diagrams present an initial linear portion followed by a plateau and a subsequent decrease of solubility. It is possible to estimate the stoichiometry of the complex from the plateau according to the expression:

(8) $$\frac{S_{T}-S_{a}}{CD_{b}-CD_{a}}$$

where $S_{T}$ is the amount of GEM added to the tubes, $S_{a}$ the maximum amount of GEM dissolved and $CD_{b}$ and $CD_{a}$ the amounts of cyclodextrin at the final and initial points of the plateau, respectively.

Finally, diagrams that present a positive deviation from linearity (A_P_ type) are associated to the formation of 1:2 substrate-ligand complexes. The stability constants $K_{1:1}$ and $K_{1:2}$ can be determined from a non-linear fit of the equation:

(9) $$S=S_{0}+S_{0}K_{1:1}\left[CD\right]+S_{0}K_{1:1}K_{1:2}{\left[CD\right]}^{2}$$

#### 3.2.4. Preparation and Characterization of Solid Dispersions

Different methods such as co-evaporation (E), coprecipitation (CP), vacuum desiccation (VD), and kneading (KN) were employed to prepare solid dispersions to assess complexation in the solid state. Physical mixtures (PM) were prepared for comparison purposes. All the systems contained 1:1 drug-CD molar ratios.

The co-evaporated (E) systems were prepared as follows: 0.125 mmol of cyclodextrin were dissolved in 10 mL of a 2:5 methanol-water solution at 55 °C, 0.125 mmol of gemfibrozil were dissolved in 12 mL of methanol, then the cyclodextrin solution was heated at 55 °C and gemfibrozil was added dropwise under continuous stirring. The solvent was subsequently eliminated by rotary evaporation at 85 °C (Buchi R-300, Flawil, Switzerland).

Solid dispersions were also prepared from a mixture containing 0.083 mol of both GEM and CD, 5 mL of 1-propanol and 10 mL of water and the solvent was eliminated by vacuum desiccation, a slower procedure than co-evaporation.

The coprecipitated systems GEM-β-CD (CP) were the solid residues obtained from the solubility experiments at the end of the plateau region of the Bs type isotherm.

The kneaded products (KP) were prepared by careful mix of 0.250 mmol of both drug and CD with a minimum volume of a 3:5 methanol:water solution. The paste obtained was kneaded for 20 min and then dried in an oven at 70 °C.

The solid dispersions were characterized using an X-ray powder diffractometer (XRD) (Bruker AXS D8 Advance, Karlsruhe, Germany) by comparison with physical mixtures prepared in the same molar ratio. The XRD patterns were collected using a Ni filter, CuKα radiation, a voltage of 40 kV and a current of 30 mA. The scanning rate was 1°/min and the time constant 3 s/step over a 2θ interval of 2–40°.

## 4. Conclusions

There are several analytical methods that can be used to study inclusion complexation with cyclodextrins both in solution and in solid state [26,27]. The well-known discrepancies between the results reported for a given host-guest system usually depend on the different physicochemical principle in which each method is based. In the present study, a reasonable agreement among the stability constants obtained by spectrofluorimetric analysis, ^1^H-NMR spectroscopy, and solubility assays was obtained, this fact supports the reliability of the values determined. The highest value corresponds to the complex of the neutral form of GEM with Meβ-CD. The relative contribution of the enthalpic and entropic effects to complexation can be related with the substituent polarity (β-CD > HPβ-CD > Meβ-CD), which gave rise to a ΔH increase together with a ΔS decrease.

Solid state complexation was evidenced by XRD analysis, which allowed us to identify different crystalline or amorphous patterns for the respective solid phases.

## Figures and Tables

**Table 1:** Quantum yields of gemfibrozil and gemfibrozil-CD systems at 25 °C.

	Φ_pH 2.8_	Φ_pH 7.0_
Gemfibrozil	0.22 ± 0.02	0.23 ± 0.01
Gemfibrozil: β-CD	0.30 ± 0.02	0.36 ± 0.01
Gemfibrozil: Mβ-CD	0.34 ± 0.03	0.36 ± 0.01
Gemfibrozil: HPβ-CD	0.33 ± 0.03	0.36 ± 0.01

**Table 2:** Binding constants and α parameter of complexes between gemfibrozil and CDs determined by fluorescence spectroscopy.

	pH 2.8		pH 7.0	
T (°C)	*K*_1:1_ 10^−2^ (M^−1^)	*a*	*K*_1:1_ 10^−2^ (M^−1^)	*a*
β-CD				
15	36 ± 2	1.45 ± 0.04	9.9 ± 0.2	1.42 ± 0.03
25	25 ± 2	1.62 ± 0.04	7.6 ± 0.3	1.58 ± 0.01
35	17 ± 1	1.81 ± 0.04	6.3 ± 0.2	1.85 ± 0.04
45	12 ± 2	2.09 ± 0.02	5.2 ± 0.2	2.30 ± 0.02
Meβ-CD				
15	42 ± 3	1.62 ± 0.03	5.1 ± 0.2	1.51 ± 0.05
25	37 ± 2	1.73 ± 0.02	4.4 ± 0.2	1.75 ± 0.06
35	33 ± 2	1.92 ± 0.03	3.8 ± 0.2	2.11 ± 0.04
45	28 ± 3	2.23 ± 0.03	3.4 ± 0.1	2.69 ± 0.09
HPβ-CD				
15	26 ± 2	1.53 ± 0.03	6.2 ± 0.2	1.51 ± 0.04
25	22 ± 1	1.63 ± 0.05	5.3 ± 0.2	1.72 ± 0.04
35	18 ± 1	1.85 ± 0.05	4.5 ± 0.3	2.02 ± 0.08
45	15 ± 1	2.22 ± 0.05	3.7 ± 0.1	2.58 ± 0.09

**Table 3:** Thermodynamic parameters of gemfibrozil complexation with different CDs.

Cyclodextrin	pH	ΔH (kJ mol^−1^)	ΔS (J mol^−1^ K^−1^)
β-CD	2.8	−26 ± 2	−22 ± 6
	7.0	−16 ± 2	−0.12 ± 2
Meβ-CD	2.8	−10 ± 1	34 ± 4
	7.0	−8.9 ± 1	19 ± 5
HPβ-CD	2.8	−14 ± 1	16 ± 2
	7.0	−13 ± 1	7 ± 2

## Data Availability

Data is contained within the article.

## References

[1] Aiassa V., Garnero C., Longhi M.R., Zoppi A. (2021). Cyclodextrin multicomponent complexes: Pharmaceutical applications. Pharmaceutics.

[2] Kurkov S.V., Loftsson T. (2013). Cyclodextrins. Int. J. Pharm..

[3] Gonzalez-Gaitano G., Isasi J., Velaz I., Zornoza A. (2016). Drug Carrier Systems Based on Cyclodextrin Supramolecular Assemblies and Polymers: Present and Perspectives. Curr. Pharm. Des..

[4] Jansook P., Ogawa N., Loftsson T. (2018). Cyclodextrins: Structure, physicochemical properties and pharmaceutical applications. Int. J. Pharm..

[5] Szejtli J., Osa T. (1996). Cyclodextrins, Vol. 3 of Comprehensive Supramolecular Chemistry.

[6] Brewster M.E., Loftsson T. (2007). Cyclodextrins as pharmaceutical solubilizers. Adv. Drug Deliv. Rev..

[7] Huang Q.P., Wang J.X., Chen G.Z., Shen Z.G., Chen J.F., Yun J. (2008). Micronization of gemfibrozil by reactive precipitation process. Int. J. Pharm..

[8] Fernández L., Martínez-Ohárriz M.C., Martín C., Vélaz I., Sánchez M., Zornoza A. (2008). Analysis of the complexation of gemfibrozil with γ- and hydroxypropyl-γ-cyclodextrins. J. Pharm. Biomed. Anal..

[9] Aigner Z., Berkesi O., Farkas G., Szabó-Révész P. (2012). DSC, X-ray and FTIR studies of a gemfibrozil/dimethyl-β-cyclodextrin inclusion complex produced by co-grinding. J. Pharm. Biomed. Anal..

[10] Fernández M.S.L., Martín C., Martínez-Ohárriz M.C., Zornoza A., Vélaz I., González-Gaitano G. Study of the interaction of β-cyclodextrins with gemfibrozil using a spectrofluorimetric method. Proceedings of the 12th International Cyclodextrin Symposium.

[11] Carrier R.L., Miller L.A., Ahmed I. (2007). The utility of cyclodextrins for enhancing oral bioavailability. J. Control. Release.

[12] Huang H., Liu F., Jia B.X., Xu K.H., Chen Z.Z., Tang B. (2007). Study on the supramolecular interaction of β-cyclodextrin with gemfibrozil by spectrofluorimetry and its analytical application. Chin. J. Chem..

[13] Maddens T., Vélaz I., Machín R., Isasi J.R., Martín C., Martínez-Ohárriz M.C., Zornoza A. (2011). Complexation of ebastine with β-cyclodextrin derivatives. J. Incl. Phenom. Macrocycl. Chem..

[14] Loftsson T., Hreinsdóttir D., Másson M. (2007). The complexation efficiency. J. Incl. Phenom. Macrocycl. Chem..

[15] Manzoori J.L., Amjadi M. (2003). Spectrofluorimetric and micelle-enhanced spectrofluorimetric methods for the determination of gemfibrozil in pharmaceutical preparations. J. Pharm. Biomed. Anal..

[16] Tang B., Jia B., Cui G., Ding Y. (2004). Study on the supramolecular interaction between β-cyclodextrin and gemfibrozil by flow injection spectrofluorimetry and its analytical application. Anal. Chim. Acta.

[17] Ross P.D., Rekharsky M.V. (1996). Thermodynamics of hydrogen bond and hydrophobic interactions in cyclodextrin complexes. Biophys. J..

[18] Saokham P., Muankaew C., Jansook P., Loftsson T. (2018). Solubility of cyclodextrins and drug/cyclodextrin complexes. Molecules.

[19] Anguiano-Igea S., Otero-Espinar F.J., Vila-Jato J.L., Blanco-Méndez J. (1997). Interaction of clofibrate with cyclodextrin in solution: Phase solubility, 1H NMR and molecular modelling studies. Eur. J. Pharm. Sci..

[20] Ding X., Zheng M., Lu J., Zhu X. (2018). Preparation and evaluation of binary and ternary inclusion complexes of fenofibrate/hydroxypropyl-β-cyclodextrin. J. Incl. Phenom. Macrocycl. Chem..

[21] Lucio D., Irache J.M., Font M., Martínez-Ohárriz M.C. (2017). Nanoaggregation of inclusion complexes of glibenclamide with cyclodextrins. Int. J. Pharm..

[22] González-Gaitano G., Sainz-Rozas P.R., Isasi J.R., Guerrero-Martínez A., Tardajos G. (2004). Site-specific interactions between 2-dibenzofuran carboxylate and β- and ϒ-cyclodextrins determined by intermolecular NOE and molecular modelling. J. Phys. Chem. B.

[23] Parker C.A., Rees W.T. (1960). Correction of fluorescence spectra and measurement of fluorescence quantum efficiency. Analyst.

[24] Connors K.A. (1987). Binding Constants: The Measurement of Molecular Complex Stability.

[25] Higuchi K.A., Connors T. (1965). Advances in Analytical Chemistry and Instrumentation.

[26] Mura P. (2014). Analytical techniques for characterization of cyclodextrin complexes in aqueous solution: A review. J. Pharm. Biomed. Anal..

[27] Mura P. (2015). Analytical techniques for characterization of cyclodextrin complexes in the solid state: A review. J. Pharm. Biomed. Anal..

